# Hepatitis B virus serum RNA transcript isoform composition and proportion in chronic hepatitis B patients by nanopore long-read sequencing

**DOI:** 10.3389/fmicb.2023.1233178

**Published:** 2023-08-14

**Authors:** Alicia Vachon, Grace E. Seo, Nishi H. Patel, Carla S. Coffin, Eric Marinier, Eduardo Eyras, Carla Osiowy

**Affiliations:** ^1^Department of Medical Microbiology and Infectious Diseases, University of Manitoba, Winnipeg, MB, Canada; ^2^National Microbiology Laboratory, Public Health Agency of Canada, Winnipeg, MB, Canada; ^3^Department of Medicine and Department of Microbiology, Immunology, and Infectious Diseases, Cumming School of Medicine, University of Calgary, Calgary, AB, Canada; ^4^EMBL Australia Partner Laboratory Network at the Australian National University, Canberra, ACT, Australia; ^5^The John Curtin School of Medical Research, ANU College of Health and Medicine, Canberra, ACT, Australia; ^6^Catalan Institution for Research and Advanced Studies, Barcelona, Spain; ^7^Hospital del Mar Medical Research Institute, Barcelona, Spain

**Keywords:** hepatitis B virus, pgRNA, serum HBV RNA, nanopore, transcript, spliced RNA, long-read sequencing

## Abstract

**Introduction:**

Serum hepatitis B virus (HBV) RNA is a promising new biomarker to manage and predict clinical outcomes of chronic hepatitis B (CHB) infection. However, the HBV serum transcriptome within encapsidated particles, which is the biomarker analyte measured in serum, remains poorly characterized. This study aimed to evaluate serum HBV RNA transcript composition and proportionality by PCR-cDNA nanopore sequencing of samples from CHB patients having varied HBV genotype (gt, A to F) and HBeAg status.

**Methods:**

Longitudinal specimens from 3 individuals during and following pregnancy (approximately 7 months between time points) were also investigated. HBV RNA extracted from 16 serum samples obtained from 13 patients (73.3% female, 84.6% Asian) was sequenced and serum HBV RNA isoform detection and quantification were performed using three bioinformatic workflows; FLAIR, RATTLE, and a GraphMap-based workflow within the Galaxy application. A spike-in RNA variant (SIRV) control mix was used to assess run quality and coverage. The proportionality of transcript isoforms was based on total HBV reads determined by each workflow.

**Results:**

All chosen isoform detection workflows showed high agreement in transcript proportionality and composition for most samples. HBV pregenomic RNA (pgRNA) was the most frequently observed transcript isoform (93.8% of patient samples), while other detected transcripts included pgRNA spliced variants, 3′ truncated variants and HBx mRNA, depending on the isoform detection method. Spliced variants of pgRNA were primarily observed in HBV gtB, C, E, or F-infected patients, with the Sp1 spliced variant detected most frequently. Twelve other pgRNA spliced variant transcripts were identified, including 3 previously unidentified transcripts, although spliced isoform identification was very dependent on the workflow used to analyze sequence data. Longitudinal sampling among pregnant and post-partum antiviral-treated individuals showed increasing proportions of 3′ truncated pgRNA variants over time.

**Conclusions:**

This study demonstrated long-read sequencing as a promising tool for the characterization of the serum HBV transcriptome. However, further studies are needed to better understand how serum HBV RNA isoform type and proportion are linked to CHB disease progression and antiviral treatment response.

## 1. Introduction

Chronic hepatitis B (CHB) infection affects over 296 million people worldwide (World Health Organization, 2022) who require monitoring of viral and biochemical markers to prevent HBV-related liver disease. While current HBV serum biomarkers inform the monitoring of CHB, there is a need for new surrogate markers to quantify intra-hepatic cccDNA levels or cccDNA transcriptional activity for clinical outcome prediction. The current gold standard method, cccDNA quantification within the liver, is challenging as it is invasive, poses risks to the patient (Crockett et al., 2006; Lin et al., 2020), and presents several technical and standardization challenges (Charre et al., 2019; Ou et al., 2020). Furthermore, HBV DNA levels are often suppressed below detectable limits during nucleos(t)ide analog (NA) treatment. Long-term monitoring of HBV DNA confirms antiviral response and adherence but is not useful for quantification of intrahepatic HBV long-term. Serum HBV RNA has been proposed as a novel surrogate biomarker of liver cccDNA levels or transcriptional activity and has been shown to be useful in various clinical contexts, such as in treatment monitoring and safe cessation (Tsuge et al., 2013; van Bömmel et al., 2015; van Campenhout et al., 2020).

The main component of secreted, encapsidated RNA is understood to be pregenomic RNA (pgRNA), which has been detected in cultured hepatocytes, liver biopsy samples from hepatitis B infected individuals, and patient serum (van Bömmel et al., 2015; Wang et al., 2016; Stadelmayer et al., 2020). In addition, several variants of pgRNA have been described: a 3′ truncated transcript resulting from a cryptic polyadenylation signal (cryptic trRNA), thought to arise from integrated HBV genomes (Hilger et al., 1991; van Bömmel et al., 2015), non-polyadenylated 3′ truncated variant transcripts, theorized to appear due to incomplete inhibition of the RNase H component of the viral polymerase during NA therapy (Zhang et al., 2016), and spliced variants of pgRNA (Bayliss et al., 2013; Betz-Stablein et al., 2016; Lim et al., 2021). HBx mRNA variants have also been described in patient serum (Stadelmayer et al., 2020).

While the clinical role of serum HBV RNA as a biomarker for monitoring CHB is highly studied, there remains a lack of understanding of the composition of serum-based RNA (Deng et al., 2022). Exploratory studies of the serum HBV transcriptome must be done to understand which HBV transcripts are encapsidated and secreted, as these remain under debate (Bai et al., 2018; Shen et al., 2020). Third-generation, or long-read sequencing of RNA using platforms developed by Pacific BioSciences and Oxford Nanopore Technologies (Oikonomopoulos et al., 2020), allows sequencing of single RNA molecules to lengths greater than the limits of short read next generation sequencing methods (>600 nt; Amarasinghe et al., 2020). Thus, long-read sequencing has been proposed as a tool to deliver a comprehensive characterization of HBV RNA species present in serum (Betz-Stablein et al., 2016) which is not possible with second-generation short-read sequencing, particularly the identification of known or novel transcript isoforms. Understanding the HBV RNA transcriptome, with matched clinical data, can provide new insights into the natural history of HBV and CHB progression (Maslac et al., 2022).

This study aimed to make preliminary observations of the serum HBV transcriptome of CHB patients having various clinical profiles and HBV genotype (gt) using Oxford Nanopore Technology (ONT) sequencing, and so provide the first detailed sequence description of serum-based HBV encapsidated transcripts.

## 2. Materials and methods

### 2.1. Patient samples

Specimens were collected from 13 HBsAg positive, CHB patients (73.3% female; 84.6% Asian; median age 30 years, range 22–41 years); all patients were under the care of a hepatologist, except one individual (19/0039) who was an asymptomatic HBsAg positive carrier. Table 1 provides the demographic, clinical and virological characteristics of patients included in the study. All patients were HBV mono-infected. This cohort, selected due to the variety of HBV genotypes and clinical/treatment circumstances available, included 2 untreated men having metabolic associated fatty liver disease (MAFLD) and 8 tenofovir disoproxil fumarate (TDF) treated pregnant females, 3 of which were longitudinally sampled post-22 weeks of pregnancy (near treatment initiation) and post-partum (LU223, LU233, and LU279; Patel et al., 2019). Most patients (10/13) were HBeAg positive and HBV gts A to F were represented within the cohort. Among treated patients with available clinical data, the mean serum ALT levels were 29.7 U/L, mean HBV viral load was 8.76 log copies/mL (near treatment initiation) and 6.35 log copies/mL (post-partum), and serum HBV RNA levels were 9.42 log copies/mL. Mean serum ALT levels, HBV viral load, and serum HBV RNA levels among the untreated patients with available clinical data were 44.5 U/L, 5.55 log copies/mL, and 7.76 log copies/mL, respectively. HBV viral load measurements were performed using the Abbott RealTime HBV Assay and converted to copies/mL (Abbott Diagnostics, Mississauga, ON; Patel et al., 2019) and serum HBV RNA levels were detected by an in-house 3’ RACE RT-qPCR assay (Vachon et al., 2022).

**Table 1:** Clinical characteristics of patients for HBV RNA long read sequencing.

Sample	Age	Ethnicity	Sex	Patient type	HBeAg status	HBV genotype	ALT (U/L)	HBV viral load (log copies/mL)	HBV RNA (log copies/mL)	Treatment
H21/3966	41	UNK	M	HBV	NEG	A	UNK	UNK	8.21	On treatment
H21/5698	22	UNK	M	HBV	NEG	A	UNK	UNK	7.92	UNK
LU205	37	Asian	F	HBV/Pregnancy	POS	B	27	8.7	10.53	TDF
LU277	33	Asian	F	HBV/Pregnancy	POS	B	34	8.6	9.59	TDF
LU373	30	Asian	M	HBV/MAFLD	POS	B	35	5.9	9.22	None
LU397	25	Asian	M	HBV/MAFLD	POS	B	54	5.2	10.22	None
LU279	24	Asian	F	HBV/Pregnancy	POS	B	44	6.9	9.95	TDF
LU279-2	24	Asian	F	HBV/Post-partum	POS	B	21	5.3	8.00	TDF
LU243	34	Asian	F	HBV/Pregnancy	POS	C	15	9.2	8.55	TDF
LU276	30	Asian	F	HBV/Pregnancy	UNK	C	20	4.5	9.46	TDF
LU233	29	Asian	F	HBV/Pregnancy	POS	C	33	9.5	9.55	TDF
LU233-2	29	Asian	F	HBV/Post-partum	POS	C	33	8.9	10.03	TDF
LU223	33	White	F	HBV/Pregnancy	POS	D	20	9.5	9.69	TDF
LU223-2	33	White	F	HBV/Post-partum	POS	D	56	4.9	9.81	TDF
19/0039	UNK	UNK	UNK	HBV	POS	E	UNK	UNK	3.85	None
291	36	Asian	F	HBV/Pregnancy	POS	F	24	8.9	9.71	TDF

UNK, unknown; ALT upper limit of normal > 35 for males, >25 for females (Coffin et al., 2018).

### 2.2. Preparation of serum RNA samples for long-read sequencing

Total nucleic acid was isolated from 600 μL of patient serum using the EasyMAG extraction system (Biomérieux Canada, Laurent, QC), with no specific enrichment for virus-like or nonenveloped particles. Twenty microgram of yeast tRNA (Life Technologies, Mississauga, ON) was added to the lysis buffer and nucleic acid elution was in 60 μL of elution buffer. Detection and quantification of RNA after serum extraction were done using the High Sensitivity RNA ScreenTape system on the 2200 TapeStation (Agilent Technologies, Mississauga, ON) using the provided protocols with the 2,200 TapeStation Software (v A.02.02 SR1).

Total extracted RNA from serum or plasma was assumed to be the fraction containing encapsidated, polyadenylated and non-polyadenylated, HBV RNA species. In order to capture possible non-polyadenylated 3′ truncated variant transcripts, a total RNA extract of each sample was also subjected to polyadenylation. Polyadenylation reactions were prepared in order to have a total volume of 40 μL (2 × 20 μL final polyadenylation reaction) in order to compensate for the additional manipulation and purification required for polyadenylation. Briefly, each 20 μL reaction included treatment of 8 μL of total RNA extracted from patient serum with an *E. coli* Poly(A) Polymerase as per the manufacturer’s protocol (New England Biolabs, Whitby, ON). Both reactions were combined and purified using Agencourt RNA Clean XP beads (Beckman Coulter, Mississauga, ON). Elution and concentration of the combined reactions was in 11 μL of nuclease-free water.

### 2.3. Direct RNA sequencing using the ONT nanopore platform

The protocol provided by ONT (Oxford, United Kingdom) for the Direct RNA sequencing kit (SQK-RNA002; version DRS_9080_v2_revP_14Aug2019) was used. Libraries were loaded on R9.4.1 MinION Flow Cells (FLO-MIN106D.8; ONT). Sequencing runs were 24 h.

### 2.4. PCR-cDNA sequencing using the ONT nanopore platform with barcoding

The protocol provided by ONT for the PCR-cDNA Barcoding kit (SQK-PCB109; version PCB_9092_v109_revD_10Oct2019) was used. The PCR step had 14 cycles of 3.5 min. Libraries were loaded on R9.4.1 MinION Flow Cells. Sequencing runs were 32 h.

### 2.5. RNA long-read sequencing error estimation

To estimate the basecall error of RNA sequenced by the ONT PCR-cDNA sequencing kit, a pgRNA transcript, transcribed from a T7 polymerase DNA vector (pGEM-T Easy, Promega Corp., Madison, WI) containing an HBV gtA 1.1x HBV genome insert (GenBank accession no. OQ54047) was polyadenylated as described previously. The polyadenylated pgRNA transcript was reverse transcribed and PCR amplified with SP6 or − 48 M13 rev primers to allow Sanger sequencing by primer walking (3 replicate reactions performed; Genbank accession nos. OQ054048-OQ054050). The polyadenylated transcripts were also sequenced using the Nanopore PCR-cDNA kit.

The combined FASTQ file from PCR-cDNA sequencing was processed in Pychopper (v 2.5.0) with default parameters and in Filtlong (v 0.2.1) using --min-length 500, −-min-mean-q 80 (percent sequence identity) and --max-length 3,400. Bowtie2 (v 2.3.4.3) was used to align the nanopore filtered read file to each of the assembled Sanger sequence contigs. The BAM2 mapping statistics tabular file output was used to assess the mean nucleotide variant percentage across approximately 3.1 kb to calculate the error rate of long-read sequencing. The mean error rate calculated among each of the three reference contigs was deemed the PCR-cDNA sequencing error.

### 2.6. Spike-in RNA variant control analysis for assessment of quality, read coverage and depth of HBV RNA long-read sequencing

Each of the 32 RNA preparations for PCR-cDNA sequencing (16 total RNA extracts and 16 polyadenylated RNA reaction products) were spiked with spike-in RNA variant (SIRV) Set-4 RNA spike-in variant mixtures (Lexogen GmbH, Vienna, Austria; Paul et al., 2016). The SIRV control consists of a mixture of RNA transcripts of various abundance and complexity, including long RNAs (4–12 kb in length). These controls allow an assessment of run performance by comparison to standardized measurement criteria. SIRV Set-4 assesses the accuracy of isoform complexity, abundance and length. For samples with high HBV serum RNA quantities (>8 log copies/mL), 1 ng of SIRV Set-4 was added. For samples with less abundant HBV RNA, 0.5 ng of SIRV Set-4 was added. SIRVsuite (Drozd et al., 2022; v 0.1.2) was used with default parameters on raw read data in order to assess the quality of the run. Coverage was assessed by evaluating coverage plot outputs for the SIRV4001, SIRV4002 and SIRV4003 isoforms, as each control transcript is approximately 4 kb in length.

### 2.7. Bioinformatic pipelines for serum HBV RNA isoform detection

**Figure 1:**
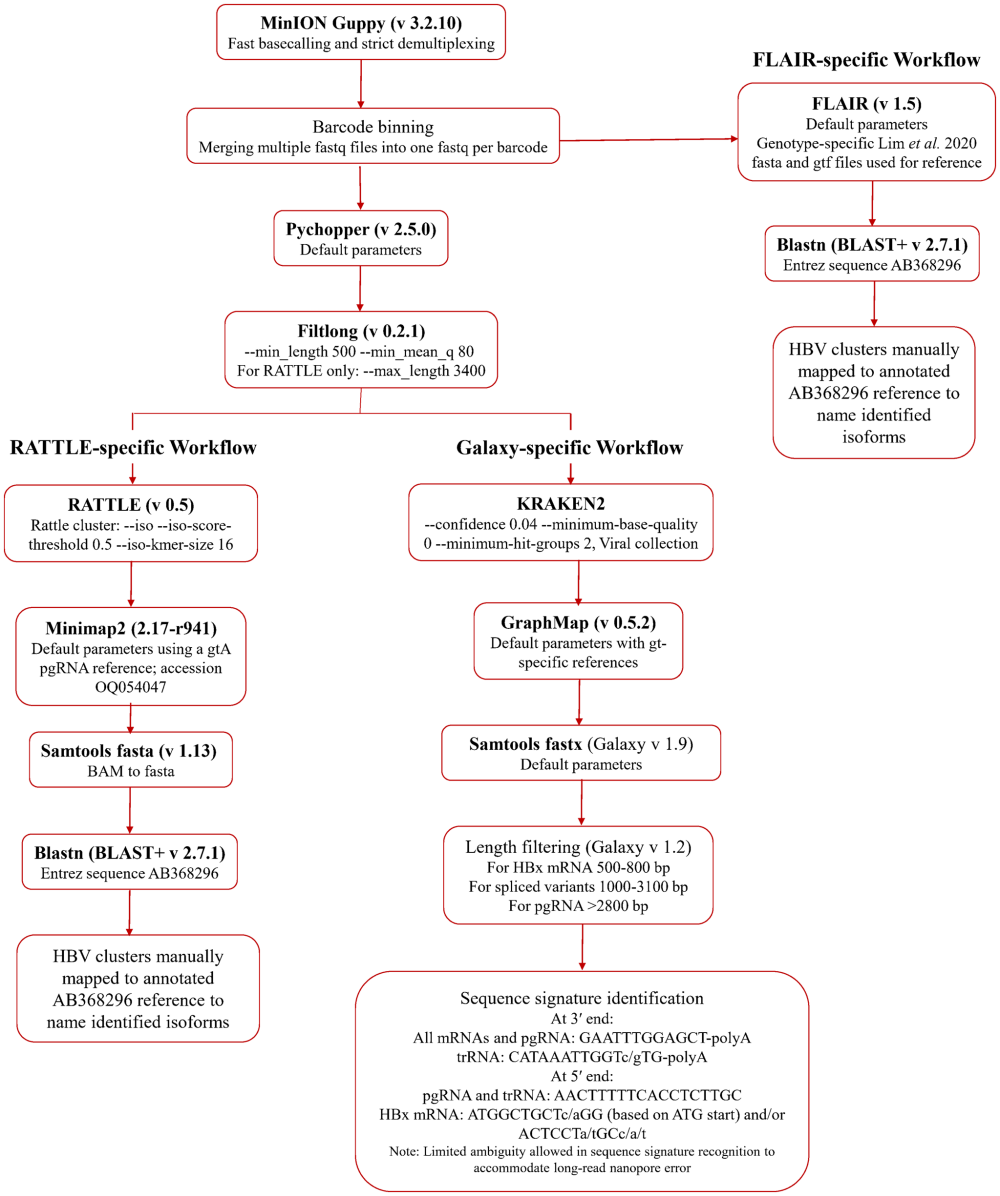
Bioinformatic methodologies for serum HBV RNA isoform identification. Basecalled, demultiplexed, and barcode binned data was used directly in the FLAIR workflow, while the RATTLE and Galaxy workflow arms used post-Pychopper and -Filtlong analyzed data. The Galaxy workflow uses Kraken 2, GraphMap, and length filtering within the online Galaxy infrastructure.

An initial quality assessment of the data was performed using NanoPlot (De Coster et al., 2018). Fast basecalling and demultiplexing were completed in Guppy (v 3.2.10, ONT). Adaptor trimming and read re-orientation were performed with Pychopper (v 2.5.0, ONT) and data was filtered by size and quality using Filtlong (v 0.2.1; Wick and Menzel, 2022), using a minimum length of 500 nt and 80 as the minimum mean quality score (expressed as sequence percent identity). Filtered data was processed for serum HBV RNA isoform identification using a reference-free method, RATTLE (de la Rubia et al., 2022), and two reference-based methods, FLAIR (Tang et al., 2020), and a GraphMap-based (Sović et al., 2016) Galaxy workflow.^1^ Transcript isoforms, identified using the three methods, were processed in blastn using an HBV genome (GenBank AB368296.1) as the Entrez Query. Identification of HBV transcripts was achieved using isoform coordinates (FLAIR and RATTLE) or specific sequence signatures (Galaxy workflow), which were manually compared to an annotated HBV genome. All data pre-processing and isoform detection pipelines are summarized in Figure 1. Data from each RNA fraction from each sample was analyzed independently using all three workflows.

#### 2.7.1. Rattle

For *de novo* isoform detection, RATTLE employs a method of read clustering based on *k*-mer comparison and matching in an iterative fashion, including error correction, and polishing to derive putative transcripts (de la Rubia et al., 2022). Prior to RATTLE analysis, the parameter of maximum length was set to 3,400 nt in Filtlong to filter out pgRNA concatemers. Quality-checked and pre-processed data was used as input in the RATTLE (v 0.5; de la Rubia et al., 2022) cluster module using --iso --iso-score-threshold 0.5 --iso-kmer-size 16 parameters. All other modules were used with default parameters. The RATTLE output transcriptome FASTQ file was aligned to an HBV gtA theoretical pgRNA reference sequence (GenBank accession no. OQ054047) to identify the HBV isoforms. Transcript proportions were determined using RATTLE-based total HBV reads.

#### 2.7.2. Flair

Raw basecalled and demultiplexed data was used as input for FLAIR (v 1.5; Tang et al., 2020). Default parameters were used. HBV gt-specific reference files that were annotated with pgRNA spliced variants from the study of Lim, et al. were used (Lim et al., 2021). Transcript proportions were determined using FLAIR-based total HBV reads.

#### 2.7.3. Galaxy workflow method

Quality-checked and pre-processed data was used as input into Kraken 2 (Wood et al., 2019; confidence 0.04, min base qual 0, hit groups 2) using the viral genomes Kraken 2 database (created 2021). The classified reads FASTQ file was then used as input for mapping (GraphMap, default parameters; Sović et al., 2016) to a gt-specific reference file containing expected HBV transcript isoforms (Supplementary Data S1.fas). This output was filtered by length according to expected transcripts. Three filters were applied: a 0.5–0.8 kb filter was applied for HBx mRNA, a 1.0–3.1 kb filter was applied for pgRNA spliced variants, 3′ truncated variants, and other HBV gene transcripts, and a filter for all transcripts over 2.8 kb was applied for cryptic trRNA and pgRNA. Each output was then manually scanned for sequence signatures to identify isoforms present in each sample and to calculate the proportion of each isoform as described above. This workflow identified full-length pgRNA as a transcript of length between 3.2 kb and 3.4 kb with the 3′ sequence having the approximate signature …GAATTTGGAGCT-poly(A) (~nt 1,922–1,935; based on GenBank AB368296), specific to the canonical HBV poly(A) signal, and the approximate 5′ sequence signature of AACTTTTTCACCTCTTGC… (~ nt 1,818–1,829). Truncated pgRNA was identified using the 5′ sequence signature and the cryptic trRNA 3′ sequence signature (…CATAAATTGGTc/gTG-poly(A), ~nt 1,788–1,801), specific to the cryptic HBV poly(A) signal. HBx mRNA, having a theoretical length between 0.5 and 0.65 kb (González et al., 2018) was identified as having the 3′-poly(A) canonical sequence signature (as above) and a 5′ sequence signature based on the X ATG start codon (ATGGCTGCTc/aGG; ~nt 1,374–1,385), falling within 100 nt of the transcript read 5′ end (González et al., 2018). These sequence signatures are conserved among all HBV genotypes. Transcript proportions were determined based on total *Hepadnaviridae* reads following Kraken 2 classification. To identify 3′ truncated variants among extracted serum RNA, the polyadenylated product of extracted RNA was sequenced and analyzed by the three pipelines. These transcripts were identified by the presence of the 5′ sequence signature and a length greater than 1.2 kb.

### 2.8. Ethics statement

The sequencing of Canadian HBV Network enrolled patient specimens at the National Microbiology Laboratory was approved by the Health Canada and Public Health Agency of Canada REB (protocol ID REB 2019-036P) and the University of Manitoba institutional ethics review board (protocol ID H2020:403).

### 2.9. Statistical analysis

Correlations between expected and measured values of SIRV control attributes were evaluated using Pearson’s correlation coefficient within SIRVsuite (Drozd et al., 2022). Kruskal Wallis ANOVA and Friedman tests were used to investigate variation in SIRV controls SIRV4001, SIRV4002, and SIRV4003, read coverage between runs and samples within a run, respectively. Statistical significance was shown by *p*-values below 0.05.

### 2.10. Data availability statement

Raw data FASTQ files were submitted to the NCBI Sequence Read Archive under BioProject ID PRJNA853798. HBV reference and pgRNA sequences used for mapping and error determination were submitted to GenBank (accession nos. OQ054047-OQ054050).

## 3. Results

### 3.1. Sequencing data quality assessment

Initial sequencing attempts using direct RNA sequencing (dRNA-seq) were unsuccessful due to the low quantity of reads mapping to HBV. An HBV gtC serum pool was extracted and concentrated to compare dRNA-seq and PCR-cDNA sequencing. Resulting FASTQ files were mapped to a theoretical pgRNA reference based on GenBank accession number AF223954 in Minimap2. While the dRNA-seq run returned 11 HBV RNA reads within the output .bam file (lengths <1.385 kb, consensus length 3.215 kb), the PCR-cDNA run returned 3,226 HBV reads (lengths <2.38 kb, consensus length 3.33 kb). Thus, PCR-cDNA sequencing was considered more effective than dRNA-seq at obtaining higher read depth for HBV RNA isoform detection from low biomass specimens.

Sixteen samples from 13 patients were subsequently processed using PCR-cDNA sequencing methods. Total RNA and a polyadenylated reaction product from each patient RNA extract (i.e., 32 RNA samples) were sequenced. The raw combined FASTQ file data for all samples had a mean read length, mean read quality (Phred score), and length N50 of 0.657 kb, 10.48, and 0.722 kb, respectively, as determined by NanoPlot analysis, which is consistent with median transcript read lengths between 0.7 and 1.8 kb reported in the literature (Bolisetty et al., 2015; Seki et al., 2019; Workman et al., 2019). The measured RNA integrity numbers for input materials were low (mean 3.69, range 2.7–5.3) however, the RNA integrity number score is based on the electrophoretic trace of cellular ribosomal RNA markers (Schroeder et al., 2006) and therefore is known to be an unreliable method to measure the integrity of RNA from serum extracts. The average sequencing error percentage (± standard deviation) based on the three reference T7-polymerase-expressed pgRNA transcripts (GenBank OQ054048-OQ054050) was 10.51% (± 6.51%). This error rate is consistent with, if not slightly below, what has been previously reported for nanopore long-read sequencing with the employed chemistry (Dohm et al., 2020; De Paoli-Iseppi et al., 2021; Sahlin and Medvedev, 2021).

The Kraken 2 standard database (created 2021-05-17) was utilized to determine reads corresponding to *Hepadnaviridae* (HBV), human (SIRV spike-in control RNA), and non-specific (other cellular, bacterial, archaea, not specified) and non-classified. Using the Kraken 2 data from all total RNA reads (i.e., not including polyadenylated product reads), the median proportion of reads for 16 samples was 0.08% for HBV, 31% for human, 49.5% for non-specific, and 19.4% for non-classified. These results, corroborated by Bracken (Lu et al., 2017) as mean proportions of HBV, human, and contaminant (calculated as 1 – [human read proportion + HBV read proportion]) reads among all total RNA samples, were 0.09, 60.4, and 39.6%, respectively. Table 2 provides the proportions of total reads used by each data analysis workflow for HBV transcript isoform identification in each RNA fraction.

**Table 2:** Proportions of HBV reads among total nanopore reads per data analysis workflow.

	Proportion of HBV reads from total reads		
Sample fraction	Galaxy	RATTLE	Flair
LU205	0.06570%	0.29815%	0.24861%
LU205-polyA	0.58257%	1.00316%	2.04270%
LU223	0.04271%	0.03687%	0.12329%
LU223-polyA	0.14506%	0.10373%	0.24327%
LU223-2	0.03173%	2.18528%	0.09622%
LU223-2-polyA	0.30060%	0.19645%	0.37299%
LU233	0.04743%	0.04992%	0.01897%
LU233-polyA	0.17223%	0.20163%	0.23629%
LU233-2	0.14287%	0.26695%	0.56282%
LU233-2-polyA	0.70281%	0.98597%	0.72642%
LU243	0.00615%	0.05136%	0.02072%
LU243-polyA	0.03086%	0.02341%	0.06916%
LU276	0.04079%	0.03710%	0.10927%
LU276-polyA	0.05097%	0.04140%	0.15076%
LU277	0.01433%	0.01378%	0.02756%
LU277-polyA	0.05860%	0.07328%	0.15687%
LU279	0.08742%	0.06986%	0.25417%
LU279-polyA	0.44644%	0.53467%	1.09711%
LU279-2	0.00097%	0.00097%	0.00437%
LU279-2-polyA	0.00314%	0.00255%	0.01255%
291	0.02291%	0.02153%	0.07372%
291-polyA	0.06450%	0.07020%	0.20791%
LU373	0.01017%	0.00751%	0.03182%
LU373-polyA	0.04942%	0.05735%	0.17091%
LU397	0.03742%	0.02943%	0.02298%
LU397-polyA	0.19680%	0.26351%	0.56014%
H21/3966	0.00904%	0.00619%	0.12139%
H21/3966-polyA	0.00471%	0.00195%	0.04875%
H21/5698	0.00052%	0.00000%	0.00278%
H21/5698-polyA	0.00294%	0.00107%	0.01050%
19/0039	0.02651%	0.01087%	0.13600%
19/0039-polyA	0.02845%	0.30064%	0.06343%

Pearson’s R values for ERCC (External RNA Controls Consortium) control isoforms generated by SIRVsuite software ranged from 0.74 to 0.88 and the mean value for all sequenced samples was 0.82. ERCC control RNA allows a measure of isoform abundance complexity, limit of detection and an assessment of workflow efficiency (Lexogen GmbH, 2021). These R values indicate a strong positive correlation between measured and theoretical concentrations of control single-isoform sequences, and therefore proportionality of HBV RNA isoforms generated during sequencing runs are also assumed to be highly accurate.

Three of the 32 RNA samples had incomplete coverage of SIRV4001, SIRV4002, and SIRV4003 (Supplementary Table S1). Low quantities of fragmented HBV reads were generated from these samples (H21/3966-polyA, H21/5698-polyA, and 19/0039-polyA). Incomplete SIRV coverage cannot be attributed to run-specific malfunction as these samples were from two different sequencing runs on two separate days. The lowest concentration of ERCC isoforms detected in all samples was 0.0000165 ng/μL (ERCC-00077, 281 nt). The lowest concentration detected in all samples in isoforms over 1.0 kb was 0.0004727 ng/μL (ERCC-00078, 1,001 nt). Variation in coverage between runs and samples within a run was assessed using the proportion of total reads mapping to SIRV4001, SIRV4002, and SIRV4003 using Kruskal-Wallis and Friedman tests, respectively. All batches (all *p* > 0.99 in Dunn’s multiple comparisons test) and samples within showed significantly similar performance, with the exception of some discrepancies observed between samples from the same run (Supplementary Table S1). Overall, coverage variation was not considered in further analyses, apart from trends in longitudinal samples.

### 3.2. Detection of serum HBV RNA isoforms

**Figure 2:**
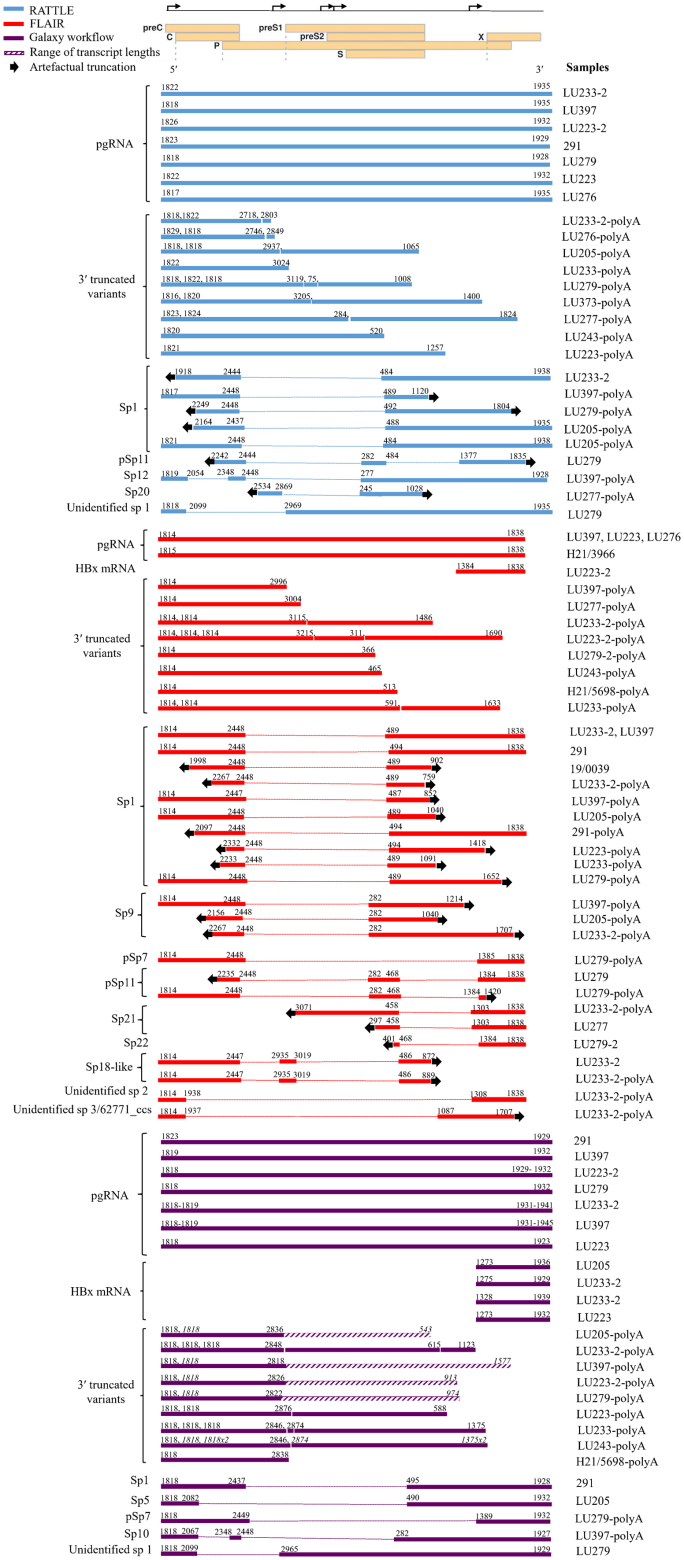
Transcript isoforms identified with RATTLE, FLAIR, and using the Galaxy workflow. Transcript coordinates are based on GenBank sequence AB368296 and exclude the poly(A) tail. Dotted lines indicate spliced sequence and arrows indicate the 5′ and 3′ ends of the sequence and the location of transcript truncation, assumed to be an artifact of sequencing. Quantities of each transcript isoform are not reflected, although proportions of each transcript type are shown in Table 3. For samples having up to three 3′ truncated variants detected, the 5′ and 3′ ends are given consecutively in order of length. For samples having more than three 3′ truncated variants detected, the 5′ and 3′ ends are denoted in italics for multiple variants and the range of variant lengths is shown by a diagonal line bar (as with the Galaxy workflow), with the shortest and longest transcript coordinates shown. Only HBx mRNAs containing all 3 sequence markers when using the Galaxy workflow are reflected. A linearized HBV genome showing reading frames (individual rectangles prefaced by the name of the open reading frame) and transcription start sites (bent arrows on the upper line denoting the complete genome) used for reference is shown at the top of the figure. Spliced variants found in poly(A) fractions were included here but excluded from further analyses.

Full-length pgRNA transcripts were considered to be those having a 3′ and 5′ end sequence approximately matching the coordinates at nt 1,934 and 1,818, respectively, of the HBV reference sequence (GenBank AB368296; gtC). All three pipelines detected full-length pgRNA transcripts within the total RNA extract (Figure 2), although FLAIR pgRNA transcripts were truncated at the 3′ end to approximately nt 1838, due to the annotated reference sequences used (Lim et al., 2021). This reference set was generated from deep RNA sequencing of HBV-infected primary human hepatocytes, with the specific goal of identifying pgRNA splice sites and determining the quantity of spliced variants; therefore, the reference sequence nucleotide coordinates impacted the isoform length identified by FLAIR. RATTLE identified full-length pgRNA transcripts with 3′ end coordinates ranging from nt 1,928–1,935 and 5′ end coordinates from nt 1,817 to 1,826 based on GenBank AB368296. The Galaxy workflow identified pgRNA 3′ end coordinates at nt 1,932 and 5′ end coordinates at nt 1,818 in nearly 50% of samples. Full-length and putative pgRNA proportions among total HBV reads tended to agree among RATTLE and FLAIR, with the Galaxy workflow often resulting in lower proportions of full-length pgRNA (Table 3). Recognition of 3′ and 5′ ends and sequence signatures was moderately elastic to allow detection of transcript ends in spite of the inherent error associated with nanopore sequencing and the possibility of nucleotide substitutions, insertions and deletions. Despite this flexibility, some HBV reads were not included within specific transcript proportions due to mapping or sequence signature requirements, and thus total proportion values may not equal 100%, particularly for the Galaxy GraphMap mapping workflow. At the time of analyses, Guppy v5 high accuracy basecalling was not available, but future transcript analyses and sequence accuracy will improve with use of the latest basecaller available.

**Table 3:** Proportions (%) of HBV transcript isoforms identified using isoform identification software.

		RATTLE	FLAIR	Galaxy	RATTLE	FLAIR	Galaxy	RATTLE	FLAIR	Galaxy	RATTLE	FLAIR	Galaxy	RATTLE	FLAIR	Galaxy
Sample	HBV geno-type	pgRNA			Putative pgRNA^*^			Spliced variants			HBx mRNA			3′ truncated variants^!^		
H21/3966	A	0.0	100.0	0.0	0.0	0.0	5.3	0.0	0.0	0.0	0.0	0.0	5.3	0.0	0.0	0.0
H21/5698	A	0.0	0.0	0.0	0.0	0.0	0.0	0.0	0.0	0.0	0.0	0.0	0.0	0.0	84.7	51.5
LU205	B	0.0	0.0	0.0	16.0	60.6	19.0	0.0	0.0	0.2	0.0	0.0	0.2	21.3	0.0	0.8
LU277	B	0.0	0.0	0.0	100.0	80.3	18.6	0.0	3.7	0.0	0.0	0.0	6.4	43.8	14.6	0.3
LU373	B	0.0	0.0	0.0	100.0	76.4	32.6	0.0	0.0	0.0	0.0	0.0	8.7	7.6	0.0	2.3
LU397	B	97.1	51.8	1.5	0.0	37.2	28.5	0.0	2.4	0.0	0.0	0.0	3.4	0.0	15.9	6.0
LU279	B	44.7	0.0	0.2	10.1	7.7	26.3	35.4	92.3	0.2	0.0	0.0	6.2	15.8	0.0	2.6
LU279-2	B	0.0	0.0	0.0	0.0	0.0	16.7	0.0	25.9	0.0	0.0	0.0	0.0	0.0	37.5	12.5
LU243	C	0.0	0.0	0.0	5.4	0.0	12.2	0.0	63.0	0.0	0.0	0.0	2.0	6.4	34.2	0.7
LU276	C	92.0	100.0	1.4	0.0	0.0	19.0	0.0	0.0	0.0	0.0	0.0	9.5	12.1	0.0	0.0
LU233	C	0.0	0.0	0.0	22.6	34.4	5.3	0.0	0.0	0.0	0.0	0.0	1.5	9.4	31.6	4.3
LU233-2	C	17.7	0.0	0.2	4.7	0.0	9.4	2.1	46.2	0.0	0.0	0.0	4.1	0.5	68.3	1.3
LU223	D	93.9	100.0	0.9	0.0	0.0	27.3	0.0	0.0	0.0	0.0	0.0	6.4	2.0	30.6	0.6
LU223-2	D	0.4	0.0	1.1	0.4	28.5	23.7	0.0	0.0	0.0	0.0	0.0	4.3	0.0	78.2	3.4
19/0039	E	0.0	0.0	0.0	100.0	0.0	15.0	0.0	8.2	0.0	0.0	0.0	0.0	0.0	0.0	0.6
291	F	7.2	0.0	0.8	77.6	0.0	18.0	0.0	73.1	0.8	0.0	0.0	9.0	0.0	0.0	0.0

^*^Putative pgRNA transcripts were defined by a length > 1.2 kb and the presence of the 3′ end pgRNA sequence signature. ^!^Classification of 3′ truncated variants are based on the polyadenylated fraction of a sample, while all other transcript classification is based on total RNA fraction.

The detection of putative transcripts within the total RNA extract was also quantified by each workflow to account for possible polymerase drop-off during nanopore library preparation or RNA degradation during extraction, although there is the possibility that putative transcripts were artefactual truncated transcripts. Putative pgRNA was defined by a length > 1.2 kb and the presence of the 3′ sequence signature. All methods produced clusters of reads (<1.2 kb) from the direct RNA extract having the expected pgRNA 5′ end, but with irregular or truncated 3′ ends. These may have been due to fragmentation or mis-priming of the non-specific poly(T) primer used for reverse transcription during library preparation (Tombácz et al., 2021), and thus <1.2 kb reads were not included in downstream analysis as they were understood to be sequencing artifacts.

A variety of pgRNA spliced variants were detected with all three methods originating from the total RNA extract and the polyadenylated products, although the Galaxy workflow, which incorporates Kraken 2, GraphMap, and length filtering tools within the online Galaxy infrastructure, was the only method providing complete spliced transcripts (Figure 2). Spliced variant transcripts were often incomplete or truncated at the 3′ or 5′ ends of sequences and thus no sequence data was available for these regions. This observation was common among FLAIR and RATTLE isoform detection, particularly among sequences from the polyadenylated extract. The observation of spliced transcript detection among a sample having undergone polyadenylation but lacking detectable spliced variant in the non-polyadenylated fraction of that sample, may have been due to concentration of the polyadenylated reaction (see Methods), allowing more sensitive sequence detection compared to the non-polyadenylated fraction for specific samples having low RNA levels. FLAIR identified at least nine pgRNA spliced variants, including Sp1, Sp9, Sp21, Sp22, pSp7, pSp11, and two novel spliced isoforms not previously described. The highest proportion of spliced variants (0–92.27%) within most patients (8/13 samples) were identified by FLAIR compared to the other two methods (5/13 using RATTLE and 4/13 using the Galaxy workflow, Figure 2), likely attributable to the spliced variant bias within the isoform reference set utilized (Lim et al., 2021). Sp1 was the most common spliced variant detected by both FLAIR and RATTLE. The identification of a novel spliced variant having breakpoints at nts 2,099 and 2,965 (or 2,969) by both RATTLE and the Galaxy workflow, in the total RNA extract from patient LU279, provided confirmation that the variant transcript was authentic and illustrated further similarity among the methods. Spliced variants found in poly(A) products were included in Figure 2, but excluded from further analyses (i.e., not represented in Table 3).

The main differences between the isoform detection methods were the identification of 3′ truncated variants and HBx transcripts. The Galaxy workflow method was the only pipeline to detect full-length HBx mRNA transcripts having variable 5′ sequence coordinates, ranging from nt 1,273 to 1,328, while FLAIR identified a transcript matching the HBx gene reference coordinates (i.e., 5′ nt 1,384 to 3′ nt 1838; Figure 2). 3′ truncated variants were observed by all isoform detection methods within the polyadenylated RNA products. The Galaxy workflow identified a large number of reads >1.2 kb and containing the 5′ sequence signature (Figure 2 and Table 3). However, no cryptic trRNA or putative cryptic trRNA, or other HBV mRNA species were identified among all samples by any isoform detection method.

### 3.3. Serum HBV RNA isoforms by HBV genotype

Sequenced samples contained HBV of genotypes A to F. All patients having available data were HBeAg positive except those infected with HBV gtA. Very few transcript isoforms were identified in gtA samples (H21/3966 and H21/5698, both sub-gt A1), most likely due to the very low proportion of HBV specific reads detected among total nanopore reads in total extracted RNA (between 0.0 and 0.12%, Table 2). We consistently observed very limited classification of HBV gtA serum RNA reads, including analysis of a gtA pool by dRNA-seq and PCR-cDNA sequencing (data not shown), in comparison to a gtC serum pool. Due to the small number of patients among each HBV genotype, it was difficult to infer other trends specific to a genotype (Table 3) and no significant associations were observed (Supplementary Figures S1–S3 showing graphical representation of the mean proportion of isoforms per genotype for each sequence analysis workflow); however, it was observed among all genotypes that full-length or putative pgRNA was the major transcript most commonly identified in total extracted RNA, dependent on the isoform identification method. Within gts B or C infected patients (5 and 3 patients, respectively), exceptions to this were observed, such that spliced variants formed the highest proportion of HBV transcripts within the total RNA extract from LU279 (gtB) and LU243 (gtC) using FLAIR. Spliced variants were also predominant among HBV reads in sample 291 (gtF). It was apparent from the genotype-specific data that FLAIR was biased toward the detection of spliced variants compared to the other two methods. The most common spliced variant identified among genotypes was Sp1 (Figure 2). Spliced variants were identified in HBV gtB, C, E, and F samples, but not in gtA and D samples, except for the polyadenylated RNA products from patient LU223 (gtD). 3′ truncated pgRNA variants within the polyadenylated RNA fraction were observed among various patients, with some representing the majority of HBV transcripts as determined by FLAIR (H21/5698, LU233-2, LU223-2).

### 3.4. Serum HBV RNA isoforms by HBeAg status

Sequenced samples were arranged into groups based on HBeAg positive or negative status. Two samples were HBeAg negative, H21/3966 and H21/5698, both HBV gtA. HBeAg positive samples comprised gtB (LU205, LU277, LU279, LU373, LU397), gtC (LU233, LU243), and gtD (LU223, LU223-2), and gtF (291). Consistent detection of specific full-length transcripts was not observed in either group, depending on the isoform identification method (Table 3). No significant difference in serum HBV RNA levels measured using 3′ RACE RT-qPCR (Table 1) was observed (*p* = 0.1143), nor was any specific trend in detected isoforms observed among HBeAg positive and HBeAg negative samples.

### 3.5. Longitudinal samples

**Figure 3:**
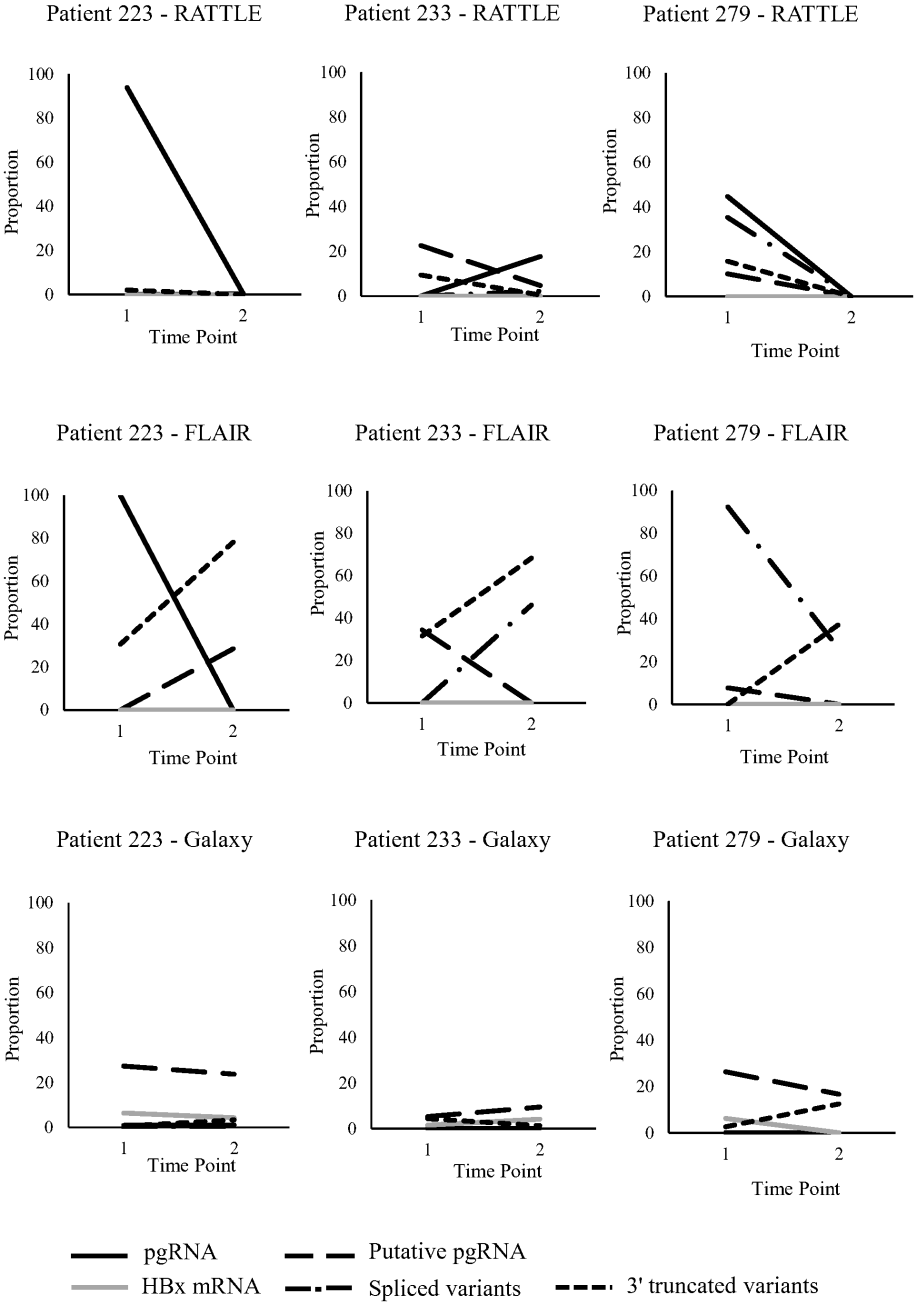
Serum HBV RNA isoforms identified in pregnant individuals sampled longitudinally. Five transcript types (full-length and putative pgRNA, HBx mRNA, spliced variants, 3′ truncated variants) were analyzed at two time points from 3 pregnant individuals; time point #1 was during pregnancy (post 22-weeks) and time point #2 was post-partum (approximately 7 months between time points).

Three patients, LU223, LU233, and LU279, were longitudinally sampled pre- and post-partum (~7 months between time points), as a preliminary investigation of serum HBV RNA isoform trends over time (Figure 3). Read counts were not normalized between samples, although trends in transcript proportions were investigated. Patients had just started treatment at the initial sampling time point, with decreasing HBV viral load observed over time, although HBV serum RNA levels were variable among the 3 patients (Table 1). General trends in transcript proportion were not consistently observed post-partum, other than an increase in 3′ truncated variants when identified by FLAIR. In general, proportional differences over time were very dependent on the method used to identify isoforms (Figure 3), and no definitive conclusions could be made due to the small number of patients and sampling time points. However, some trends were consistent among RATTLE and FLAIR for certain isoforms (i.e., pgRNA for patient 223, putative pgRNA for patient 233, spliced variants for patient 279).

## 4. Discussion

Serum HBV RNA has been shown to be a valuable surrogate biomarker of intra-hepatic cccDNA levels in monitoring of CHB infection. More specifically, it has been used to monitor response to treatment (van Campenhout et al., 2020), to predict viral relapse after NA cessation (Tsuge et al., 2013), and to identify CHB phases of infection (Liu et al., 2019). While many studies have described the clinical role of serum HBV RNA, less is known about the proportion, composition and natural history of HBV RNA transcripts in the serum. It has been proposed that a better understanding of this could inform CHB clinical management and predict natural history (Betz-Stablein et al., 2016; Bai et al., 2018; Shen et al., 2020; Maslac et al., 2022). To our knowledge, this study is the first to employ nanopore PCR-cDNA sequencing to identify transcript isoforms and their approximate proportions present in the serum HBV transcriptome of multiple genotypes of HBV.

### 4.1. pgRNA was the most frequent and most abundant transcript detected

Pregenomic RNA was repeatedly detected among samples (15/16; 93.8%), either as a transcript of full-length (~3.33–3.34 kb) or a partial transcript termed “putative” as it contained the pgRNA 3′ sequence signature but was of variable length > 1.2 kb. pgRNA has been described to comprise the greatest proportion of serum HBV RNA (Günther et al., 1997; Wang et al., 2016; Lim et al., 2021). Both RATTLE and the Galaxy workflows identified full-length pgRNA in half of the samples tested, while FLAIR and RATTLE most accurately represented pgRNA at high proportions within total HBV reads. This is most likely due to the ability of FLAIR and RATTLE to cluster short reads into larger isoforms (Tang et al., 2020; de la Rubia et al., 2022). Putative pgRNA transcripts were included in the isoform analysis to account for the possibility of polymerase drop off during PCR-cDNA transcript amplification, RNA fragmentation, or transcript strand pore blocking. A common observation with nanopore sequencing is a coverage bias toward the transcript 3′ end, including with cDNA sequencing (Amarasinghe et al., 2020), thus putative or potential pgRNA were included in the analysis, although the possibility of coverage bias of fragmented RNA also exists.

A striking observation from our HBV isoform identification data is the absence of cryptic trRNA. This transcript isoform, having a 3′ truncation due to the presence of a cryptic polyadenylation signal upstream of the canonical poly(A) site, was firstly observed in tumor and non-tumor tissue from patients with HCC (Hilger et al., 1991). It is thought to be derived from the transcription of integrated HBV genomic material and its 3′ end coordinate is estimated to be around nt 1,680 of the HBV genome (Hilger et al., 1991; Schutz et al., 1996). While it has been suggested that cryptic trRNA is typically present in patients in more advanced disease states, including HCC, due to the observed higher frequency of HBV integration during these phases, methodological limitations, such as PCR bias toward highly expressed transcripts (De Paoli-Iseppi et al., 2021), could have resulted in the lack of cryptic trRNA identification. The relatively young age and predominance of HBeAg seropositivity of the cohort may also be associated with a reduced level of integrated hepatitis B. Moreover, FLAIR and RATTLE have the ability to collapse truncated reads into larger isoforms (Tang et al., 2020; De Paoli-Iseppi et al., 2021; de la Rubia et al., 2022), which may have affected the ability to identify cryptic trRNA by these methods. Regardless, transcripts having the cryptic trRNA 3′ sequence signature were also not detected with the Galaxy workflow using the cryptic poly(A) site sequence signature. The detection of insignificant cryptic trRNA transcript levels has also been reported from cultured cell lysate extracts (Ng et al., 2023).

Following synthetic polyadenylation of serum HBV total extracted RNA, pgRNA 3′ truncated variant transcripts were identified in 14 of 16 clinical samples. These transcripts contain the 5′ sequence signature of pgRNA transcripts but are not full length, with variable length greater than 1.2 kb. The 3′ truncated variant transcript proportion of HBV reads within polyadenylated RNA products varied depending on the isoform detection method, but comprised the majority of transcripts identified in several specimens. Indeed, a 3′ truncated variant was the sole transcript detected in one specimen (H22/5698; gtA), as identified by both FLAIR and Galaxy workflows. It has been reported that many HBV RNA species secreted in serum are non-polyadenylated (Shen et al., 2020), with pgRNA 3′ truncated variants first identified by Zhang et al. in hepatoma cells (Zhang et al., 2016). These variants were thought to arise from RNase H cleavage of the 3′ end of pgRNA due to incomplete inhibition of the HBV polymerase by DNA polymerase inhibitors (Zhang et al., 2016). Therefore, it is expected that these transcripts are present in patients treated with NAs, which was observed, although not consistently, possibly due to the short treatment duration for most of the cohort (pregnant patients). Alternatively, the observation of variable length transcripts having the pgRNA 5′ sequence signature may be due to initial amplification mis-priming (i.e., not at the true polyadenylation site) or pgRNA fragmentation during nucleic acid isolation or the polyadenylation reaction. In a human hepatocyte cell line treated with lamivudine, the 3′ end of pgRNA 3′ truncated variants most often occurred between nts 1,726 and 1,802, ranging from nts 1,373 to 1,802 (Zhang et al., 2016), resulting in transcripts with approximate lengths between 2,770 and 3,200 nucleotides. Very few of the identified 3′ truncated variants observed by the isoform detection methods utilized had these expected coordinates (1 by RATTLE and 3 by FLAIR). However, a different study described clones of 3′ truncated variants to have 3′ coordinates at nt 1,243, 2,579, 2,622, and 3,182 by 3′ RACE RT-PCR, while encapsidated HBV RNA was detected to be as short as a few hundred nucleotides (Bai et al., 2018). Therefore, it is difficult to conclude whether the 3′ truncated variants identified are true biological occurrences or sequencing artifacts.

An interesting observation was the myriad of pgRNA spliced variants identified with all three isoform detection methods, including Sp1, Sp5-6, Sp9-10, Sp12, Sp20-22, pSp7, and pSp11 (Günther et al., 1997; Bayliss et al., 2013; Chen et al., 2015; Betz-Stablein et al., 2016; Lam et al., 2017; Lim et al., 2021; Ng et al., 2023). Additionally, some spliced variants resembling previously identified isoforms but whose splice junctions were unidentified, were detected by the isoform tools employed. FLAIR identified two novel spliced variants, “unidentified sp2” and one that was very similar to the 62,771 ccs spliced variant described by Lim et al. (2021). FLAIR also identified an Sp18-like, or 66,956 ccs, spliced variant (Lim et al., 2021). A novel spliced variant (“unidentified sp. 1”) was detected by both RATTLE and the Galaxy workflow analysis. This finding appears to support the inference that the annotated reference set used with FLAIR will influence the detection and quantification of isoforms (Amarasinghe et al., 2020). Spliced variants have been reported to compose a minor proportion of wild type HBV RNA in serum (Günther et al., 1997; Bayliss et al., 2013; Lam et al., 2017). Our data showed that FLAIR, in conjunction with the particular annotated reference used for analysis, was more likely to report high proportions of spliced variants. If detected with FLAIR, spliced variant proportions ranged from 2.44 to 92.27%, whereas RATTLE and the Galaxy workflow reported spliced variant proportions up to 35.4 and 0.8%, respectively. In concordance with previous reports in the literature, Sp1 is the most common spliced variant in patient serum (Günther et al., 1997; Chen et al., 2015); however, other reportedly abundant variants (Chen et al., 2015; Lam et al., 2017; Lim et al., 2021), such as Sp2, Sp3, Sp13, and Sp14, were not detected in our dataset. While this perhaps speaks to non-sufficient depth of our sequencing, it has been shown using FLAIR that spliced variant detection differences are mostly dependent on genotype and experimental conditions and not as dependent on the depth of sequencing (Lim et al., 2021). An exception to this discordance is the abundance of Sp9 as the second most abundantly identified spliced variant by FLAIR analysis, which concurred with the Lim et al. study (Lim et al., 2021). The presence of archetypal spliced pgRNA variants within polyadenylated products, albeit without fully present 5′ and/or 3′ ends, lends support to the observed 3′ truncated variants, and thus may represent analytical rather than biological fragmentation of the polyadenylated products during preparation or sequencing.

HBx full-length transcripts having a reported length between 0.7 and 0.9 kb (Kobayashi et al., 1997; Chen et al., 2015) with 5′ end coordinates between nt 1,278 and 1,350, have also been described in patient plasma and cell culture (Stadelmayer et al., 2020), although the transcript 5′ end coordinates reported were highly variable, suggestive of truncated HBx transcript forms. The presence of non-canonical HBx transcripts described by Stadelmayer et al. (2020) based on cloned 5′RACE RNA amplicons from three HBeAg positive patients were not explored among the patient samples in this study, and thus may have been missed. In this study, HBx mRNA transcripts were only identified by the Galaxy workflow. These transcripts had 5′ ends between nt 1,273 and 1,328, indicating a canonical HBx mRNA. The 5′ end of HBx mRNA identified with FLAIR was nt 1,384, likely due to the annotation used in the reference set (Lim et al., 2021).

The use of a SIRV control allowed for measurement of accuracy, precision, and inter-experiment comparability (Paul et al., 2016), the latter of which was especially useful due to the multiple sequencing runs performed in this study. It is recommended that SIRV spike-in quantities be between 1 and 10% of total RNA sequencing input (Hardwick et al., 2016; Lexogen GmbH, 2021); however, others have used a fixed SIRV quantity per experiment (de la Rubia et al., 2022), which is the approach taken in this study. Thus, it is somewhat expected that SIRV reads will make up the majority of reads given that 0.5–1.0 ng of spike-in RNA was added to study samples, irrespective of total RNA concentration, and given the low biomass of RNA that is expected in serum extracts (Zhou et al., 2019; Jia et al., 2021). Indeed, HBV RNA is expected to comprise a very small fraction of the total serum RNA, due to encapsidated HBV RNA secreted as a replicative intermediate through a “non-classical” pathway (Wang et al., 2016). Enrichment of HBV-specific RNA using probe capture may solve this issue, as it has been shown to increase the abundance of transcript templates in cultured cell lysate extracts for sequence analysis (Ng et al., 2023).

### 4.2. Genotypic and clinical observations with identified HBV serum RNA transcripts

Previous studies have looked at differences in serum HBV RNA by HBV genotype and have found that gtB, C, D, and F expressed higher levels of pgRNA, whereas gtA expressed the lowest (Elizalde et al., 2021). Similarly, a higher diversity of pgRNA spliced variants was found in gtB and C (Maslac et al., 2022), and the highest proportions of serum HBV RNA spliced variants have been observed in gtC and D (Chen et al., 2015). No spliced variants were detected in the two gtA samples in this study, but gtB and C samples had the highest proportions and the most diversified pgRNA spliced variants (Figure 2 and Table 3). This suggests that spliced variant generation may be genotype-specific, but also corroborates the more severe clinical presentation of HBV infections with gtC, as spliced variant expression is linked to liver disease pathogenesis (Chen et al., 2015; Maslac et al., 2022). It is of note that gtA, and sub-gtA1 in particular, is also associated with rapid progression of chronic disease (Bannister et al., 2020; Elizalde et al., 2021); however, since patients in the gtA group in this study were all HBeAg negative, serum HBV RNA levels in these patients were expected to be lower than in the HBeAg positive patients (van Bömmel et al., 2015).

Patient sample RNA transcripts were analyzed based on HBeAg serostatus. Very few studies have evaluated serum HBV RNA levels and profiles as a function of HBeAg presence or ALT elevation (Maslac et al., 2022); however, it is critical to understand serum HBV RNA type and quantity throughout chronic infection to better inform clinical decision-making regarding treatment and disease state, particularly when quantifying the analyte in serum. Only two HBeAg-negative patients were included in this study (all gtA), which was a limitation in understanding the association of HBeAg status and HBV RNA isoforms. Spliced variants isoforms were observed in just over a third of HBeAg-positive patients, by at least one sequence analysis workflow. As spliced variant presence is associated with viral replication (Chen et al., 2015), it would be expected that these variant transcripts may be present in a greater proportion in a phase characterized by increased viral load (HBeAg-positive). Furthermore, HBeAg-positive individuals with high antigen and viral load levels are characterized by greater T cell exhaustion (Kennedy et al., 2012), and potential impairment of immune cell recognition of spliced variants and their expressed proteins. Although spliced variant prevalence in the liver has been shown to increase with advancing liver disease (Betz-Stablein et al., 2016), no cohort patients had known cirrhosis or HCC, and young, pregnant patients are unlikely to have fibrosis. Indeed, two post-partum patients having FibroScan results at the time of specimen collection showed normal levels (LU223-2, 4.5 kPa; LU233-2, 3.8 kPa; normal range is 2–7 kPa), suggesting a lack of liver disease progression.

Longitudinal samples were collected from three NA-treated individuals pre- and post-partum to allow preliminary observations of serum HBV RNA over time. Very few studies have looked at the dynamics of HBV RNA composition and quantity in clinical samples, but it is known that spliced variant diversity increases over time (Betz-Stablein et al., 2016). No significant differences in read coverage among longitudinal samples was observed; however, significant changes in relative serum HBV RNA isoform levels over time could not be determined, primarily due to the small number of study patients and consecutive samples collected. Based on FLAIR isoform detection alone, an increased proportion of 3′ truncated variants was observed among all pregnant people from pregnancy to post-partum (Figure 3). Considering that the appearance of 3′ truncated variants is associated with NA treatment (Zhang et al., 2016), this observation may be due to the initiation of NA therapy during pregnancy or possible immune reconstitution post-partum. A decrease over time in HBx mRNA proportion with the Galaxy workflow was observed in two of three patients. While it has been observed that HBx mRNA production decreases after the establishment of HBV infection (Guan et al., 2022), the role of the expression levels of this transcript in chronic infection is still unclear. Future studies should focus on HBV RNA isoform level dynamics over time using an increased number of patients having greater consecutive sampling to overcome the limitation of the current study. Validation of findings by PCR-based methods (Bayliss et al., 2013), short-read deep sequencing (Lim et al., 2021), or Northern blotting of HBV transcripts from clinical samples (Bai et al., 2018) may also confirm or expand on the present observations.

### 4.3. Challenges and limitations of serum-based viral transcriptomic identification

HBV full genome sequencing studies, or those exploring the HBV transcriptome, have largely employed PCR- or amplicon-based deep sequencing methods (Betz-Stablein et al., 2016; Sauvage et al., 2018; McNaughton et al., 2019; Astbury et al., 2020; Lim et al., 2021; Matsuda et al., 2021; Maslac et al., 2022). To date, PacBio long-read sequencing has been used to analyze the HBV transcriptome within cultured cells (Ng et al., 2023), but there is only one account of ONT sequencing of HBV nucleic acid extracted directly from patient plasma without the use of PCR amplification (Colson et al., 2021); however, very few HBV reads were obtained. While PCR-free RNA sequencing methods present the least bias (Chen et al., 2021; De Paoli-Iseppi et al., 2021), they have been shown to be unsuitable for samples with low target RNA quantities and high rates of fragmentation (Vacca et al., 2022). PCR-cDNA sequencing has been shown to produce shorter reads with more depth than dRNA-seq (Chen et al., 2021), and consequently the PCR-cDNA method was chosen to sequence the clinical samples in this study. PCR-cDNA long-read sequencing has been used to study various viral transcriptomes, including human cytomegalovirus (Balázs et al., 2017), herpesviruses (Tombácz et al., 2019; Kakuk et al., 2021), and poxviruses (Tombácz et al., 2021). PCR-cDNA long-read sequencing uses a feature selection method by employing a poly(T) primer for reverse transcription, along with full transcript-length PCR using global amplification primers (Oikonomopoulos et al., 2020). Studies have shown that the complexity of viral transcriptomes is consistently underestimated by standard methods and long-read sequencing is a valuable tool to better characterize viral transcripts (Kakuk et al., 2021).

There were distinct differences in the isoforms detected and their proportionality depending on the analysis pipeline used. FLAIR and RATTLE are mostly used for transcript identification in eukaryotic cells and RNA spike-in material (Al Kadi et al., 2020; Tang et al., 2020; de la Rubia et al., 2022), and their effectiveness in the classification of viral transcripts has yet to be assessed. RATTLE uses a reference-free clustering algorithm which has been shown to have the same accuracy in isoform identification and quantification as reference-based methods (de la Rubia et al., 2022). As this study aimed to detect and discover serum-based transcripts, the use of a *de novo* isoform identification method was important. However, RATTLE often mis-identified the expected pgRNA 5′ sequence signature due to HBV having two direct repeats, DR1 (nt 1,825–1,834) and DR2 (nt 1,590–1,600), employed during HBV replication. The pgRNA “true” 5′ end is associated with DR1, and thus, recognition of the upstream DR2 site created RATTLE-identified transcripts that were longer than expected, having a 5′ end at approximately nt 1,590. RATTLE tends to merge very similar reads into a single transcript and is thus more conservative in terms of the number of predicted known or novel transcripts identified.

While RATTLE’s precision was found to be lower than reference-based methods such as FLAIR and StringTie (de la Rubia et al., 2022), we observed that most of the spliced variants identified by RATTLE were previously described. Although these results are encouraging, there is less confidence for isoforms with low read counts (de la Rubia et al., 2022). The preliminary observations of this study would be strengthened by validation of isoform detection and quantification in an HBV infection model, as well as the use of standard and quantitative PCR to confirm splice donor and acceptor sites and spliced variant proportions described in the current study. The Galaxy workflow identified much fewer transcripts than RATTLE and FLAIR based on isoform proportionality, although it was most effective at identifying full-length transcripts at single-read resolution. Potential limitations of the Galaxy GraphMap workflow are the reliance on visual transcript identification using set 5′ and 3′ sequence signatures for specific transcripts, which is laborious and error-prone, such that true transcripts may be missed. Validation of each method to accurately identify and characterize expected transcripts using a curated panel of known HBV transcripts and RNA quantity should help delineate the most appropriate method for characterization of the HBV serum transcriptome within patients. As this was an exploratory and observational study, we find that RATTLE analysis was the preferred method of the 3 workflows. This finding is based on ease of use, and unbiased identification of transcripts and spliced variants. The availability of more complete annotated HBV reference files for FLAIR analysis should also expand the suitability of that workflow.

The fact that FLAIR requires an annotated reference file posed a challenge, as no annotated HBV genomes with all expected transcript isoforms exist in present databases. The choice of reference highly influences the efficacy of transcript identification by FLAIR, which was the most effective method for the identification of known and novel spliced variants. Validation of splice junction sites, such as comparison to short read Illumina data (Tang et al., 2020) was not used; however, FLAIR and RATTLE perform splice site or error correction, respectively, and therefore have improved isoform identification accuracy (De Paoli-Iseppi et al., 2021). RATTLE contributes to this through a polishing step of iterative clustering to remove errors or non-consensus reads from contigs/alignments to resolve the final consensus cluster (de la Rubia et al., 2022). Despite error correction, we still observed variation in transcript boundary coordinates that were most likely due to insertion/deletion errors observed with nanopore sequencing (Amarasinghe et al., 2020; Delahaye and Nicolas, 2021) as opposed to a biological difference. However, the higher error rates attributed to nanopore sequencing are not necessarily a limitation for isoform identification, as the overall transcript can be identified by well annotated 5′ and 3′ ends of known transcripts (Tombácz et al., 2020).

While evaluating the long-read sequencing data, the presence of co-morbidities and the clinical status of the HBV-infected patient is important to consider. The primary purpose of serum HBV RNA quantification is for clinical applications; however, the composition and proportion of each serum RNA species may differ depending on the clinical profile (Lim et al., 2021; Deng et al., 2022). The immune state or other host contributions to disease progression will affect HBV replication and transcription from certain HBV promoters (Asadi-Asadabad et al., 2021; Ibrahim et al., 2021), although the total serum HBV RNA level may remain stable. Diagnosis of MAFLD in HBV infected individuals has been shown to increase the risk of severe liver-related disease (Wong et al., 2014), while pregnancy impacts maternal immunity during CHB infection (Gao et al., 2021). The HBV RNA composition and transcript proportionality were observed to differ during and after pregnancy in this study, although serum HBV RNA levels remained relatively stable over time (Table 1), similar to other findings during pregnancy (Patel et al., 2019). Further study is needed to better understand the association among clinical and treatment profiles and the presence of HBV RNA transcripts.

### 4.4. Conclusion

These results present for the first time, initial observations of the serum-based HBV transcriptome by long-read sequencing technology among longitudinal and cross-sectional specimens of patients having various hepatitis B clinical profiles and genotypes. Pregenomic RNA was observed to be the major transcript detected among patients infected with various HBV genotypes (A to F), although the proportions of serum-based HBV transcripts detected by cDNA-PCR nanopore sequencing differed dependent on the sequence analysis workflow. The results obtained from long-read sequencing of the HBV transcriptome illustrated the strengths and challenges of using nanopore long-read technology and the utility of several bioinformatics methods for serum HBV RNA isoform sequencing and quantification. The study provided novel insights into the profiles and proportions of HBV RNA isoforms in chronic infection to support further understanding of this biomarker for management of CHB patients.

## Data availability statement

The datasets presented in this study can be found in online repositories. The names of the repository/repositories and accession number(s) can be found at: NCBI - PRJNA85379.

## Ethics statement

The studies involving human participants were reviewed and approved by the Health Canada and Public Health Agency of Canada REB (protocol ID REB 2019-036P) and the University of Manitoba institutional ethics review board (protocol ID H2020:403). Written informed consent for participation was not required for this study in accordance with the national legislation and the institutional requirements.

## Author contributions

CO and AV: conceptualization, formal analysis, funding acquisition, methodology, and writing. GS, AV, NP, and EM: data acquisition. CO: project administration and supervision. AV, GS, and CO: result analysis and interpretation. CO, CC, EM, and EE: laboratory and software resources. All authors reviewed, revised, and approved the manuscript.

## Funding

AV was supported by a 2020 Canadian Liver Foundation Graduate Studentship.

## Conflict of interest

The authors declare that the research was conducted in the absence of any commercial or financial relationships that could be construed as a potential conflict of interest.

## Publisher’s note

All claims expressed in this article are solely those of the authors and do not necessarily represent those of their affiliated organizations, or those of the publisher, the editors and the reviewers. Any product that may be evaluated in this article, or claim that may be made by its manufacturer, is not guaranteed or endorsed by the publisher.

## Footnotes

^1^ usegalaxy.org
