# Supplementary material for: Hepatitis B virus serum RNA transcript isoform composition and proportion in chronic hepatitis B patients by nanopore long-read sequencing
Source: Front Microbiol. 2023 Aug 14;14:1233178. doi: 10.3389/fmicb.2023.1233178 (PMC10461054; doi:10.3389/fmicb.2023.1233178)
Supplement: Supplementary file 1 [file Data_Sheet_1.zip › Supplementary Figures Legend.docx]

**Supplementary Figures**

**Figure S1.** Mean proportion of transcripts identified by RATTLE analysis for each genotype. The proportion of HBV transcript isoforms (Table 3) was averaged among patients of each genotype.

**Figure S2.** Mean proportion of transcripts identified by Flair analysis for each genotype. The proportion of HBV transcript isoforms (Table 3) was averaged among patients of each genotype.

**Figure S3.** Mean proportion of transcripts identified by Galaxy analysis for each genotype. The proportion of HBV transcript isoforms (Table 3) was averaged among patients of each genotype. Discontinuous Y axes were used to show mean proportions of pgRNA, spliced variant RNA and 3ʹ truncated variant RNA due to the very low mean proportions among each genotype. The five transcripts identified by Galaxy analysis are shown.
