# Supplementary material for: Hepatitis B virus serum RNA transcript isoform composition and proportion in chronic hepatitis B patients by nanopore long-read sequencing
Source: Front Microbiol. 2023 Aug 14;14:1233178. doi: 10.3389/fmicb.2023.1233178 (PMC10461054; doi:10.3389/fmicb.2023.1233178)
Supplement: Supplementary file 1 [file Data_Sheet_1.zip › Supplementary Table S1.docx]

Table S1: SIRV4001, SIRV4002, and SIRV4003 coverage for each sample used for long read sequencing

|  |  |  | SIRV4001 | | SIRV4002 | | SIRV4003 | | ANOVA result per batch |
| --- | --- | --- | --- | --- | --- | --- | --- | --- | --- |
| Sample | Batch | Total reads | total reads | proportion | total reads | proportion | total reads | proportion |  |
| LU205 | 1 | 656049 | 37 | 0.00564% | 90 | 0.01372% | 147 | 0.02241% | p=0.0281 |
| LU205-polyA | 1 | 255593 | 27 | 0.01056% | 77 | 0.03013% | 57 | 0.02230% |  |
| LU233-2ǂ | 1 | 760817 | 83 | 0.01091% | 157 | 0.02064% | 287 | 0.03772% |  |
| LU233-2-polyA | 1 | 542107 | 28 | 0.00517% | 67 | 0.01236% | 125 | 0.02306% |  |
| LU397ǂ | 1 | 713614 | 27 | 0.00378% | 61 | 0.00855% | 94 | 0.01317% |  |
| LU397-polyA | 1 | 664118 | 34 | 0.00512% | 69 | 0.01039% | 151 | 0.02274% |  |
| LU223-2ǂ | 2 | 586150 | 85 | 0.01450% | 132 | 0.02252% | 294 | 0.05016% | p=0.0018 |
| LU223-2-polyAǂ | 2 | 371592 | 16 | 0.00431% | 34 | 0.00915% | 70 | 0.01884% |  |
| 291 | 2 | 580574 | 62 | 0.01068% | 120 | 0.02067% | 267 | 0.04599% |  |
| 291-polyA | 2 | 333315 | 17 | 0.00510% | 25 | 0.00750% | 68 | 0.02040% |  |
| LU279 | 2 | 569695 | 44 | 0.00772% | 123 | 0.02159% | 170 | 0.02984% |  |
| LU279-polyA | 2 | 244824 | 31 | 0.01266% | 49 | 0.02001% | 107 | 0.04370% |  |
| LU223 | 3 | 805430 | 85 | 0.01055% | 159 | 0.01974% | 288 | 0.03576% | p=0.0001 |
| LU223-polyA | 3 | 617000 | 32 | 0.00519% | 70 | 0.01135% | 159 | 0.02577% |  |
| LU233 | 3 | 843399 | 138 | 0.01636% | 213 | 0.02525% | 453 | 0.05371% |  |
| LU233-polyA | 3 | 285670 | 22 | 0.00770% | 45 | 0.01575% | 59 | 0.02065% |  |
| LU277 | 3 | 1088706 | 176 | 0.01617% | 205 | 0.01883% | 401 | 0.03683% |  |
| LU277-polyA | 3 | 988058 | 43 | 0.00435% | 57 | 0.00577% | 165 | 0.01670% |  |
| LU243 | 4 | 796336 | 89 | 0.01118% | 97 | 0.01218% | 229 | 0.02876% | p=0.0005 |
| LU243-polyA | 4 | 469892 | 25 | 0.00532% | 38 | 0.00809% | 77 | 0.01639% |  |
| LU276 | 4 | 541788 | 83 | 0.01532% | 126 | 0.02326% | 225 | 0.04153% |  |
| LU276-polyA | 4 | 417869 | 16 | 0.00383% | 29 | 0.00694% | 68 | 0.01627% |  |
| LU373 | 4 | 452499 | 57 | 0.01260% | 76 | 0.01680% | 199 | 0.04398% |  |
| LU373-polyA | 4 | 530107 | 26 | 0.00490% | 50 | 0.00943% | 69 | 0.01302% |  |
| LU279-2ǂ | 6 | 617220 | 90 | 0.01458% | 127 | 0.02058% | 305 | 0.04942% | p=0.0174 |
| LU279-2-polyA | 6 | 509832 | 36 | 0.00706% | 78 | 0.01530% | 152 | 0.02981% |  |
| H21/5698 | 6 | 576308 | 72 | 0.01249% | 75 | 0.01301% | 187 | 0.03245% |  |
| H21/5698-polyAǂ | 6 | 1123639 | 3 | 0.00027% | 9 | 0.00080% | 23 | 0.00205% |  |
| H21/3966ǂ | 8 | 210070 | 26 | 0.01238% | 64 | 0.03047% | 118 | 0.05617% | p=0.0028 |
| H21/3966-polyAǂ | 8 | 615335 | 7 | 0.00114% | 14 | 0.00228% | 36 | 0.00585% |  |
| 19/0039 | 8 | 377211 | 39 | 0.01034% | 81 | 0.02147% | 160 | 0.04242% |  |
| 19/0039-polyA | 8 | 551821 | 6 | 0.00109% | 17 | 0.00308% | 34 | 0.00616% |  |

ǂ samples within same batch that had significantly different SIRV coverage (p<0.05)
