# Supplementary figures and images for: Hepatitis B virus serum RNA transcript isoform composition and proportion in chronic hepatitis B patients by nanopore long-read sequencing
Source: Front Microbiol. 2023 Aug 14;14:1233178. doi: 10.3389/fmicb.2023.1233178 (PMC10461054; doi:10.3389/fmicb.2023.1233178)

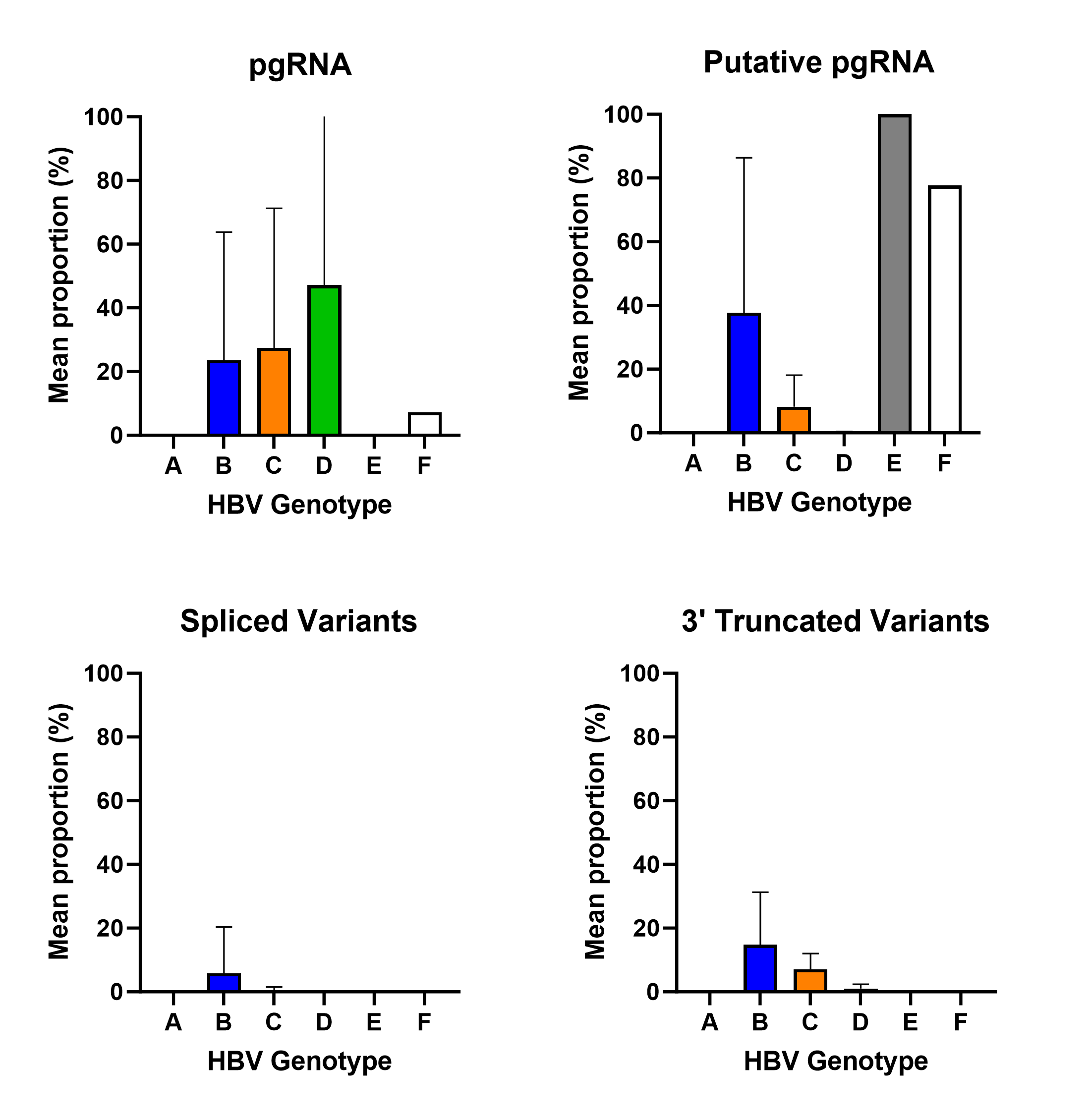

Supplement: Supplementary file 1 [file Data_Sheet_1.zip › Figure S1.tif]

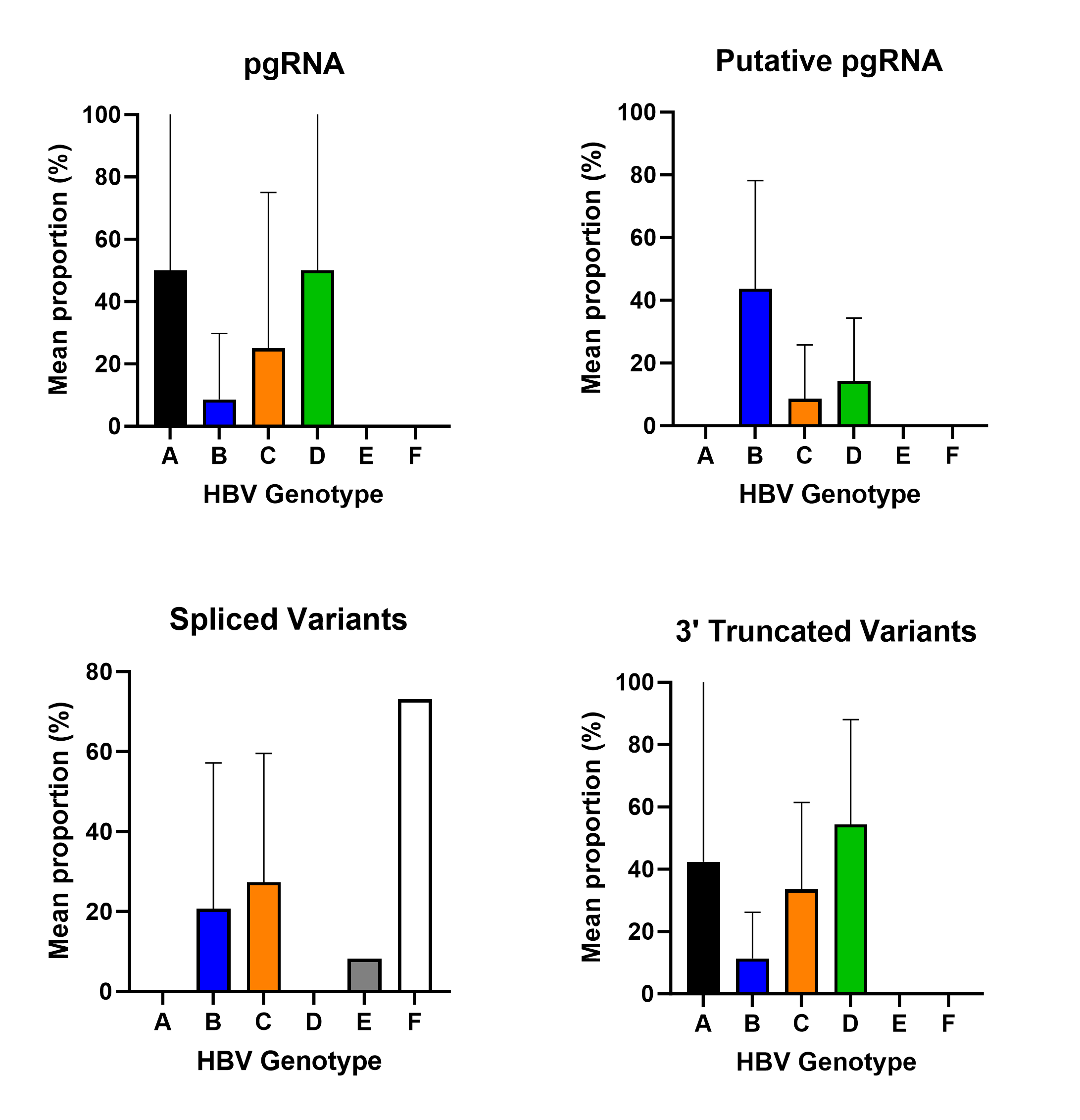

Supplement: Supplementary file 1 [file Data_Sheet_1.zip › Figure S2.tif]

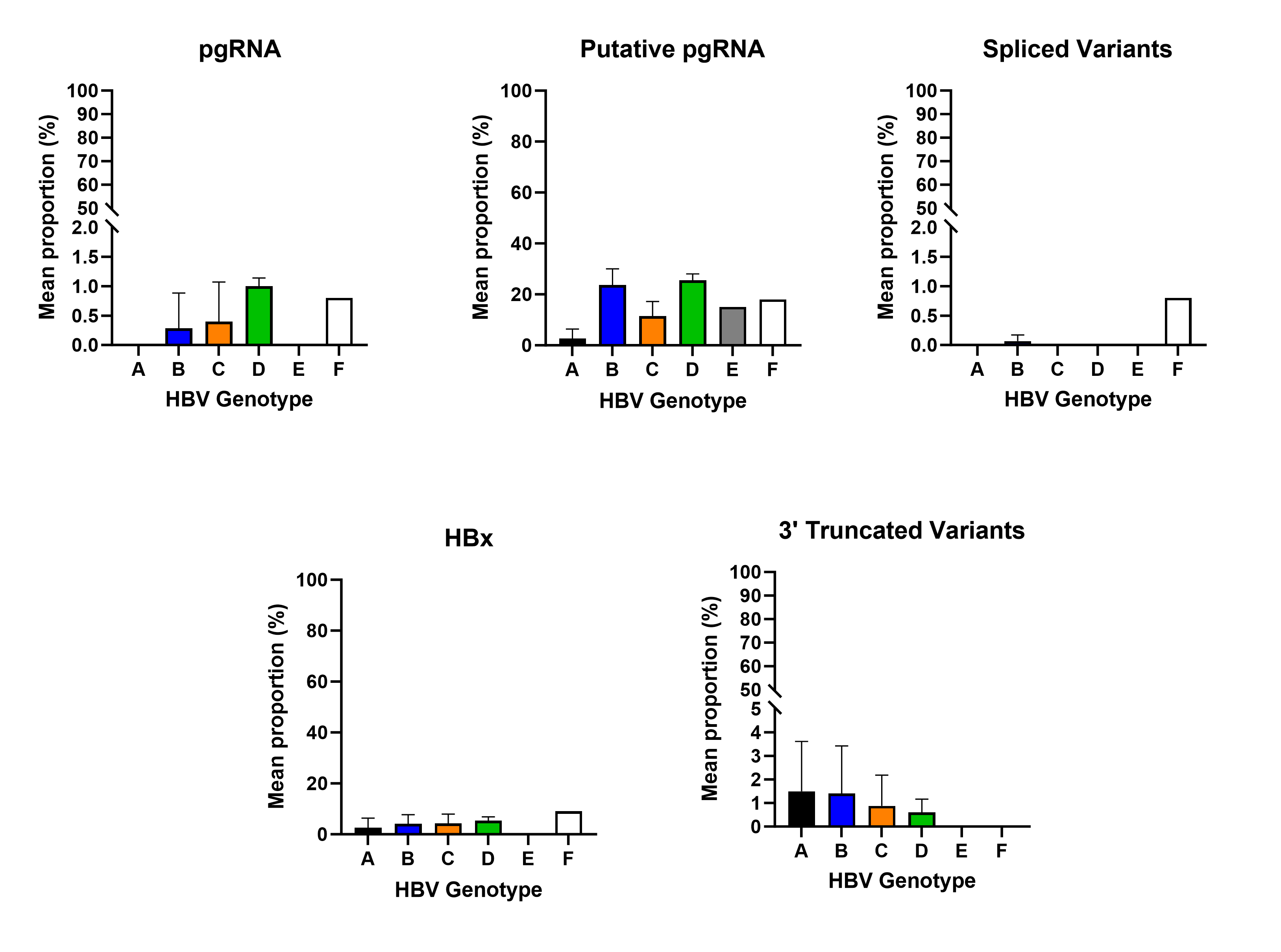

Supplement: Supplementary file 1 [file Data_Sheet_1.zip › Figure S3.tif]
